# Furthering the Evidence of the Effectiveness of Employment Strategies for People with Mental Disorders in Europe: A Systematic Review

**DOI:** 10.3390/ijerph15050838

**Published:** 2018-04-24

**Authors:** Amalia Muñoz-Murillo, Eva Esteban, Carolina C. Ávila, Klemens Fheodoroff, Josep Maria Haro, Matilde Leonardi, Beatriz Olaya

**Affiliations:** 1 Research, Innovation and Teaching Unit, Institut de Recerca Sant Joan de Déu, Esplugues de Llobregat, 08830 Barcelona, Spain; jmharo@pssjd.org (J.M.H.); beatriz.olaya@pssjd.org (B.O.); 2 Parc Sanitari Sant Joan de Déu, Universitat de Barcelona, Fundació Sant Joan de Déu, Sant Boi de Llobregat, 08830 Barcelona, Spain; 3 Department of Medical Information Processing, Biometry and Epidemiology (IBE), Chair for Public Health and Health Services Research, Research Unit for Biopsychosocial Health, Ludwig-Maximilians-Universität (LMU), 81377 Munich, Germany; eva.esteban@med.lmu.de; 4 Department of Psychiatry, Universidad Autónoma de Madrid and CIBER of Mental Health (CIBERSAM), 28049 Madrid, Spain; carolina_avila@hotmail.com; 5 Gailtal Klinik—Neurologische Rehabilitation, A-9620 Hermagor, Austria; klemens.fheodoroff@gailtal-klinik.at; 6 Instituto de Salud Carlos III, Centro de Investigación Biomédica en Red de Salud Mental (CIBERSAM), 28029 Madrid, Spain; 7 Neurology, Public Health and Disability Unit, Neurological Institute C. Besta IRCCS Foundation, 20133 Milan, Italy; matilde.leonardi@istituto-besta.it

**Keywords:** systematic review, effectiveness, job access strategies, return to work, mental health, Europe

## Abstract

(1) Purpose: This systematic review aims to assess the effectiveness of strategies used in the professional (re)integration of persons with mental disorders (MD) in European countries; (2) Methods: We conducted a search for scientific publications available in relevant electronic databases (Medline, PsycINFO, CDR-HTA, CDR-DARE, and Cochrane Library). The present study collected evidence on the effectiveness, from 2011 to 2016, of employment strategies for persons with MDs; (3) Results: A total of 18 studies were included, representing 5216 participants (aged 18–65, mean age of 38.5 years old) from 7 countries. Job access programs demonstrated effectiveness in four out of six studies. Return to work (RTW) interventions showed significant positive results in two studies, while four studies did not refer to effectiveness. There were inconsistent results in another four studies; (4) Conclusions: Our findings highlight the complexities of the implementation of employment strategies (job access and return to work). Job access strategies seem to improve employment outcomes. The effectiveness of return to work strategies remains unclear. The involvement and commitment of physicians, employment specialists, and employers, and employees capacity for self-care seem decisive for employment re-integration success. Further analyses are needed to assess the cost-effectiveness of these interventions and corroborate our results, with longer follow-ups.

## 1. Introduction

Recent studies estimate that disability resulting from mental illness is responsible for 32% of global years lived with disability (YLDs) and 13% of global disability-adjusted life years (DALYs) [1,2]. Indeed, in terms of DALYs, depression causes 6% of all disease burden in Europe [3]. Four of the six leading causes of YLDs are neuropsychiatric disorders worldwide (depression, alcohol use disorders, schizophrenia, and bipolar disorders) [3], with the human, social, economic, and employment costs of mental disorders (MD) considered to be high [1,4]. Poor education, unemployment, and social inequalities are commonly associated with mental disorders [5], and people suffering from mental illness usually suffer from high levels of disability and comorbidity [6,7,8]. In 2004, the economic cost of depression in Europe was estimated at 118 billion Euros and has been found to be the most costly European brain disorder, corresponding to 33% of the total cost [9]. Therefore, mental disorders are still considered one of the priorities to be addressed thoroughly by national and European social and economic policy makers [4,10].

Inactivity and unemployment rates are significantly higher among persons with MDs, compared with those without [11,12,13]. Based on data from the 2011 ad hoc module of the EU Labour Force Survey, the employment rate in EU-28 for persons with limitations at work caused by a health condition was about 30% less than for people without such limitations [14]. In fact, the amount of productive time lost at work due to personal or family health reasons is likely to be higher in workers suffering from depression than in workers without the disorder [15]. 

Several factors might explain the higher rate of unemployment in persons with mental illness. For instance, stigma [16,17], non-discriminatory workplaces, and limited access to specific support [18,19] among employers can make it more difficult for people with MD to obtain a job. Depressed employees’ symptoms have been found to be related to work absence and lower productivity [20]. Indeed, mental health problems might affect work performance by causing fatigue and cognitive deterioration [21,22]. Additionally, about half of long-term absences at work are caused by MD [23,24]. Long-term absences seem to increase the difficulties these workers have when returning to work [25,26].

Although work can be a stressor for people with mental disorders, it seems that the benefits of working outweigh its detrimental effects. In a review by Waddell and Burton in 2006 [27], it was found that the lack of work among people with mental disorders was strongly associated with a higher number of consultations with the general practitioner than for the general population. Persons who had been unemployed for more than 12 weeks showed higher rates of depression and anxiety and the rates of suicide were higher among persons who were unemployed. The authors found that return to work led to improvements in health and wellbeing, and for people who were ill or disabled, placement in work improved their health status. Additionally, they reported that the health status of people who move off welfare benefits also improved. They concluded that work could be beneficial for health and wellbeing and that these benefits were also applicable to people with MD. Therefore, the implementation of employment interventions in this type of population is especially indicated to reduce the burden associated with mental disorders. 

Employment integration interventions for unemployed people are divided into two groups, here: traditional vocational rehabilitation models [28] and the supported employment model (SE) [29]. These models represent what we have called “job access strategies”. Traditional models focus on the interventions in the setting prior to initiating work activity. They can include, among other elements, prevocational training, clubhouse, or sheltered workshops. Conversely, SE focuses on the immediate competitive job search. The SE method appears to be effective in gaining employment for people with MD [30,31,32]—it has been proved to be more effective than other vocational training programs [33,34] and it may reduce feelings of exclusion and mental illness stigma [35,36]. Individual Placement and Support (IPS) is one of the most structured and properly methodized SE programs to date [37,38]. Available evidence of the effectiveness of employment strategies shows that IPS is more effective than traditional models of vocational rehabilitation [39,40,41] and this effectiveness was found across diverse cultural and economic backgrounds [40,41]. 

Other types of employment (re)integration interventions are focused on persons with chronic diseases (PwCD) with a job and on sick leave due to health conditions. In our review, these are grouped as return to work (RTW) strategies for persons with MD. These models focus on interventions for employees on sick leave due to mental health problems. These programs aim to get employees back to work in some capacity as soon as possible. They can include part-time sick leave interventions, absenteeism prevention, and making accommodations, if necessary.

Recently, Europe has taken up the integration of PwCD challenge. Studies on the health care burden, the increasing prevalence of chronic diseases, aging, and social inequalities are being conducted to find innovative and sustainable solutions [42]. Different strategies have been implemented at local, regional, and national level in several European countries [43]. Previous reviews assessing the effectiveness of employment integration and (re)integration strategies are focused on the USA and worldwide [38] or centered on specific strategies (i.e., IPS). However, there is still a need for a comprehensive review of the effectiveness of these types of interventions for people with mental disorders in a European setting. This review is part of the EU-funded Participation to Healthy Workplaces and Inclusive Strategies in the work sector Project (PATHWAYS; www.path-ways.eu). Pathways aims to contribute to the European dialogue concerning the development of strategies and further recommendations for promoting the participation of PwCD, including MD, in the labor market. To assess the evidence of the employment strategies for PwCD, a comprehensive systematic review was carried out in PATHWAYS for a wide range of chronic conditions, including mental disorders (see Sabariego et al., 2018 [44]) as well as another review focused on qualitative studies [45]. Our interest was focused on the effectiveness of employment interventions within the European framework aiming at improving access to (competitive) work, return to work, and job maintenance for people with MD of working age.

## 2. Materials and Methods

**Figure 1:**
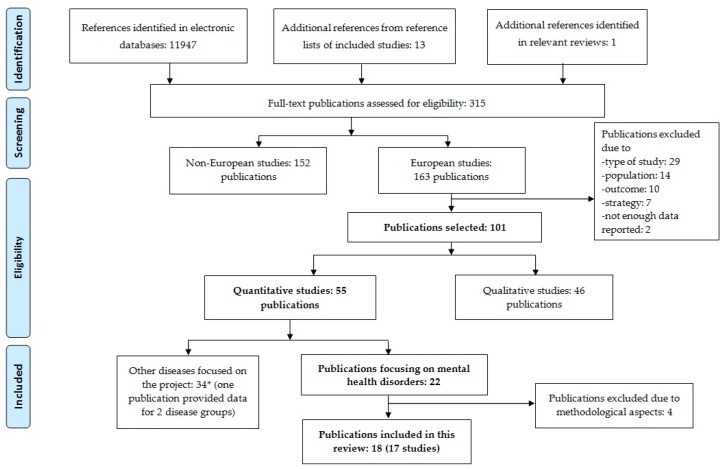
Flow chart of the systematic review carried out in the PATHWAYS project. Boxes in bold highlight the flow chart of the present review, assessing employment strategies for persons with MD.

We searched for scientific publications on this topic in relevant electronic databases (Medline, PsycINFO, CDR-HTA, CDR-DARE, and Cochrane Database of Systematic Reviews). Searches were run in April 2016. Additionally, for papers not identified in the electronic search, we examined references from included papers and from recent relevant employment strategy reviews. Figure 1 shows the general search strategy used within the Pathway project. It is meaningful to mention that the original systematic review run within the scope of the PATHWAYS project included different categories of disease—such as mental disorders, musculoskeletal disorders, cancer, and neurological, metabolic, respiratory, and cardiovascular diseases—and different study designs [44]. This paper focuses on the results of parts of this systematic literature review, particularly the effectiveness of strategies for integration and re-integration to work for persons with MD. Results of other parts of this systematic review are reported in several publications of this special issue.

This review was conducted in accordance with the standard procedure recommended by Cochrane [46]. The search terms for the PATHWAYS systematic review are described in Supplementary Materials. The syntax for Medline was adapted from Clayton et al. [47]. Duplicates were deleted and the references were screened and selected.

### 2.1. Selection Criteria

Studies were eligible for inclusion if they:

(a) were published between January 2011 and April 2016;
(b) were published in English;
(c) were intervention studies—namely, randomized trials, nonrandomized controlled trials, noncontrolled pre-post intervention studies;
(d) were observational studies—namely, cohort studies, case–control studies, cross-sectional studies, descriptive longitudinal studies;
(e) were qualitative studies or mixed-methods studies (for present study only);
(f) were carried out in the 28 countries of the European Union, in Norway, Lichtenstein, Iceland, or Switzerland, or in non-European countries with western lifestyle: Canada, United States of America, Australia;
(g) reported on effectiveness regarding at least one of the following work outcomes:
  (1) employment status (employed/unemployed);
  (2) return to work;
  (3) absenteeism (sick leave);
  (4) maintaining a job;
  (5) obtaining a job;
(h) investigated variables potentially affecting effectiveness (e.g., views and experiences of involved persons with a given strategy).

Regarding the target population, studies were included if they focused on the working population aged 16–65 years. Regarding health conditions, studies were included if they focused on:

(a) PwCDs in general (i.e., specific conditions are not further specified in the studies or results for different conditions are reported together) and persons with disabilities in general;
(b) the following disease groups: mental disorders, musculoskeletal disorders, and cancer, neurological, metabolic, respiratory, and cardiovascular diseases;
(c) the following specific diseases: depression, back and neck pain, migraine, diabetes mellitus, chronic obstructive pulmonary disease, and ischemic heart disease.

Studies were excluded if they:

(a) included participants with mainly other chronic diseases than the ones defined above;
(b) included participants aged <16 or >65 years;
(c) were case report/case series, psychometric studies, letters, comments, editorials, overviews without empirical primary or secondary data, reviews (systematic and nonsystematic reviews, health technology assessments) and meta-analyses, protocols, studies reporting exclusively on design or baseline data;
(d) considered no effectiveness outcomes—for example, studies reporting only on costs resulting from the implementation of strategies—nor variables potentially affecting effectiveness;
(e) did not focus on a concrete strategy or group of strategies, for example, studies focusing on factors facilitating return to work after sick leave in general;
(f) were published in languages other than English;
(g) were published before 2011;
(h) had no abstract available.

### 2.2. Study Selection and Data Extraction

The selection of the abstracts retrieved was based on the inclusion and exclusion criteria and it was performed by trained reviewers. An independent second reviewer double checked approximately 30% of the references. In case of discrepancy, agreement was reached through discussion based on the information available in the title and abstract. Finally, full versions of papers considered eligible were retrieved and examined by two researchers. 

### 2.3. Study Quality Assessment

The quality of the included studies was assessed with quality appraisal checklists for quantitative intervention studies: National Institute of Health and Clinical Excellence (NICE) in the UK [48]. Methodological Assessment for the Complete PATHWAYS Review has been published elsewhere [44].

Regarding effectiveness, we answered the question on whether data supported the effectiveness of the strategy with four categories:

− Yes. Yes was selected if estimates for relevant work outcomes had an adequate *p*-value, usually <0.05, or if the confidence interval for the estimate excluded the no-effect value (e.g., the value 1 was not included in the confidence interval of reported odds ratio);
− Unclear. Unclear was selected if the precision of the effect estimate was not reported, or if results were inconsistent or difficult to interpret (e.g., statistically nonsignificant but large estimates in subgroup analyses);
− No. No was selected if data did not support the presence of an effect of the intervention on relevant work outcomes.

As described above, the searches performed in the PATHWAYS project had a wider target. This manuscript focuses on quantitative studies evaluating the effectiveness of interventions carried out in European countries among persons with MD.

The results of the present study are reported following the PRISMA statement [49]. Data synthesis will be presented according to type of integration and (re)integration interventions (job access and RTW).

## 3. Results

### 3.1. Literature Search

The search performed in PATHWAYS retrieved a total of 11,947 references. Figure 1 shows the flowchart for the studies’ selection process. Boxes in bold highlight the flowchart of the present review focused on quantitative papers assessing strategies for persons with mental disorders.

After assessing the full-text European articles, 62 papers were excluded because of the study design, target participants (i.e., younger, older, or not representative), outcome criteria (i.e., not related to work), type of strategy (i.e., not focused on the effectiveness of a program or work outcomes), and not enough data reported (i.e., the results were not clearly reported or there were significant concerns regarding drop-out rates). One hundred one publications were selected and 55 quantitative studies were included. Of these, a total of 22 European studies focused on effectiveness of employment strategies for persons suffering from MD were finally selected. The studies were conducted in Belgium (1), Denmark (5), Germany (1), Italy (1), Netherlands (10), Norway (3), Sweden (12), Switzerland (2), UK (7), and other European studies (2).

A further assessment of the full-text articles resulted in 17 studies meeting our inclusion criteria for the present review [50,51,52,53,54,55,56,57,58,59,60,61,62,63,64] as well as 1 study with a longer follow-up of a prior publication [65]. Regarding the quality assessment, the study of Germundsson et al. (2012) [32] was eventually included because of the qualitative assessment reported. Eighteen studies were finally included in synthesis.

### 3.2. Study Characteristics

The total sample represents 5216 people with MD, with a mean age of 38.5 years old, and 42.3% were men. The mean duration of the studies was 17.8 months. Table 1 summarizes the characteristics of the selected studies. Seven out of the 18 studies were based on job access and 11 out of the 18 studies were RTW-approach strategies. Supplementary Materials provide a short description of each intervention program from the different studies included in this review.

### 3.3. Intervention Analyses

Table 2 summarizes the results according to the type of intervention (job access and RTW).

#### 3.3.1. Job Access Programs

A total of seven studies were based on job access interventions. In one study [50], time to first job was reduced by 218 days and mean hours worked per week increased by 9 hours in the IPS group. The authors of the study reported that participants receiving IPS achieved competitive employment (24.9% vs. 14.3%) compared to pre-IPS participants. However, significant differences were not reported. High attrition rates were also reported: 71.4% in the control group and 52.2% in the IPS group. Bejerholm et al. [56] evaluated IPS in Sweden using a randomized control trial (RCT) with a 12-month follow-up. In this study, the majority (90%) of the IPS participants were involved in work, internship, or academic project, whereas solely the 24% of the traditional vocational rehabilitation (TVR) group achieved the aforementioned activities. The authors also found that 6 months after the program began, there was no difference between groups in terms of employment achievement. At 18 months, the rate of competitive employment, the number of weeks and hours worked, and work tenure were all greater in the IPS group, compared to the TVR group (46% vs. 11%; difference 36%, 95% CI = 18–54%). Therefore, IPS was found to be more effective for gaining employment than TVR. Another IPS study in the Netherlands [60] found that the proportion of participants who found competitive jobs at 6 months was not significant (44% of IPS participants found competitive work, compared with 25% of participants supported by TVR). However, job access at 18 and 30 months was significantly higher in the IPS group than in the comparison group, as were the mean hours worked in competitive jobs. Only one IPS study [58] included in this review showed relatively low rates of competitive employment in both the intervention group (IPS) and the traditional vocational services group, although significantly more participants randomized in the treatment group obtained competitive employment (22% vs. 11%, *p* = 0.041). Additionally, this study suggested that participants with previous work experience within the last 5 years were more likely to attain competitive employment. In 2- and 5-year follow-up IPS studies developed in Switzerland [59,65], participants in the IPS group were more likely to obtain competitive work than those in traditional vocational rehabilitation, worked more hours and weeks, earned higher wages, and had longer job tenures. In the first part of the study [59], the results showed that working competitively at the end of 2 years was relatively stable for both groups over the next 3 years (45% for IPS and 15–17% for TVR). At a 5-year follow-up [65], results showed significant group effect (*p* < 0.001) and a significant time effect (*p* < 0.001). Thus, IPS was more successful than TVR (65% vs. 33%, *p* = 0.002) regarding competitive employment rates, length of employment, total and annual weeks in competitive work, job tenure in longest competitive work, and mean hours worked.

As for vocational rehabilitation (VR) based on supported employment programs, one study [32] compared three different factors: employment status, disposable income, and sum of allowances. They reported significant changes in the SE groups between baseline and 2-year follow-up (*p <* 0.001). These results suggested that individuals who received the SE intervention were hired faster, earned a higher disposable income, and had lower individual allowances than participants who were not engaged in the program. The control group showed significant changes solely for the sum of allowances measures (*p* = 0.003). 

#### 3.3.2. RTW Programs

Eleven studies were based on RTW programs. We found two RTW studies focusing on sickness absence. The first one evaluated part-time sick leave (PTSL) in the Netherlands using a register-based cohort study [66]. The authors reported inconclusive results for the intervention group. Thus, PTSL had no effect on the duration until returning to regular working hours for employees with mental disorders, and did not reduce time of RTW in this cohort. However, duration of sick leave for employees with other health conditions was significantly reduced. Similarly, another cohort PTSL study was conducted in Sweden [51]. In common with the previous study, persons in the PTSL program were compared to persons in full-time sick leave for one year. In this study, full recovery of lost work capacity from the participants assigned to PTSL was less likely than in the full-time sick leave (FTSL) group. Additionally, they found that the probability of full recovery of lost work capacity at the beginning of PTSL instead of FTSL was relatively low (0.015) when PTSL was assigned at the beginning of the intervention, but relatively high (0.387) and statistically significant when assigned after 60 days of FTSL. 

Another study evaluated work-focused cognitive-behavioral therapy (W-CBT) in the Netherlands [53]. In this controlled trial, over 90% of participants from both groups (CBT as usual and W-CBT) recommenced work within one year, although the W-CBT participants achieved this result about 2 months earlier. Partial return to work was found more often in the W-CBT group and occurred 12 days earlier, and fewer steps were performed to full RTW than in the CBT as usual (2.94 vs. 4.26; F(1, 147) = 16.72, *p* ≤ 0.01). Overall, W-CBT participants were more likely to achieve full (HR = 1.56, *p* ≤ 0.05, SE = 0.19) and partial RTW (HR = 1.59, *p* ≤ 0.05, SE = 0.20), suggesting a shorter duration of the intervention until both full and partial RTW. However, more relapses in the return to work process were observed in the W-CBT, but this difference was not statistically significant. The authors concluded that CBT showed significant effects on duration until RTW in favor of the work-CBT group. Another similar controlled trial study [52] evaluated work-related CBT in Germany. Both CBT as usual and work-related CBT significantly reduced days of incapacity to work. Consistent with the previous study, more W-CBT participants were involved in employment activities at 1-year follow-up than CBT as usual (13 vs. 8 employees, *p* = 0.039). Moreover, the reduction of days of incapacity to work was larger in work-related CBT. 

One study evaluating a web-based intervention in the Netherlands in an RCT [64] showed unclear results for this intervention. The authors underlined a significant effect for duration until first RTW at 77 days in the care as usual (CAU) group (IQR = 29.0–152.3) and at 50 (IQR = 20.8–99) days in the intervention group (HR = 1.39, 95% CI = 1.03–1.87, *p* = 0.03). The authors found a significant effect of the web-based intervention for duration until first return to work only, and no significant effect for time to full return to work and number of days of sickness absence.

In a 12-month follow-up RCT study in the Netherlands [55], problem-solving interventions delivered by occupational physicians were found to be effective in reducing the incidence of recurrent sickness absence, compared with care as usual (adjusted OR = 0.40, 95% CI = 0.20–0.81). The intervention group had a median of 365 days (IQR = 174–365) to recurrent sickness absence and the CAU group had a median of 253 days (IQR 117–365; *p* = 0.003). However, the analysis of the authors suggested that the effect of the problem-solving intervention on recurrent sickness absence did not significantly differ at the three follow-up measurements.

An exposure-based return to work program provided by occupational physicians, in addition to CAU, was also evaluated in a 12-month follow-up study in the Netherlands [61]. Exposure-based RTW (RTW-E) participants needed more time to achieve full RTW (209 days; 95% CI 62–256) compared to workers receiving CAU (153 days; 95% CI 128–178) and the difference was significant (*p* = 0.02). However, regarding time to partial return to work and number of sick-leave relapses, the differences between the groups were not significant. 

Another collaborative care treatment was also assessed in the Netherlands [63]. In this 12-month follow-up RCT, participants receiving collaborative care needed less time to return to work (with a difference of 2.8 months between groups) and fewer days on sick leave than the comparison group, but these differences were not statistically significant.

In Denmark, a multidisciplinary, coordinated and tailored return-to-work intervention for both groups of employed and unemployed persons with common mental disorders was developed [54]. The intervention program was shown to exacerbate time to RTW (HR = 0.50; 95% CI = 0.34–0.75). These results determined a rather negative effect and delayed return to work compared to conventional case management, after accounting for confounders. Remarkably, after 1 year, more participants of the coordinated intervention were receiving sickness absence benefits than the conventional case management recipients. 

An 18-month follow-up RCT was performed in the Netherlands [57]. This study suggested that additional occupational therapy (OT) did not significantly improved work participation among participants. In the course of the intervention, participants receiving the additional OT were more likely to get back to work when depression symptoms remitted (adjusted group difference = −1.9, 95% CI = −19.9 to 16.2) compared to the treatment as usual (TAU) group. However, those in TAU + OT increased probability of long-term RTW in good health (OR = 1.9, 95% CI 1.1 to 3.2, *p* = 0.02) in contrast with the treatment as usual participants. Nevertheless, hours of absenteeism were significantly decreased in both groups with no difference between them. Additionally, there were no differences in full or partial RTW between the intervention and the TAU group. 

#### 3.3.3. Mixed Programs (Job Access and RTW)

An 18-month follow-up RCT study in Norway, combining interventions focused on job access and RTW in the same intervention, evaluated a systematic and integrated approach including CBT and, if needed, IPS [62]. The intervention group increased or maintained their work participation at follow-up compared to the control group (44.2% vs. 37.2%, *p* = 0.015). The difference remained significant after 18 months (difference 7.8%, *p* = 0.018), and was greater for participants receiving long-term benefits (difference 12.2%, *p* = 0.007). 

## 4. Discussion

The aim of our study was to review the effectiveness of employment strategies in European countries regarding persons with mental disorders. Overall, our findings suggest that the implementation and effectiveness of job access strategies are shown to be effective, while the results of the included papers studying RTW strategies are inconclusive.

We determined that four out of six papers analyzing IPS effectiveness [50,58,59,65] found it to be effective at the first time-point measurement and the other two studies [56,60] also found IPS to be effective at 18-month follow-up. Our findings support the effectiveness of IPS for improving job access and return to work among persons suffering from mental illness. Similarly, IPS effectiveness has been demonstrated in the USA [31,67] and worldwide [41].

In our review, one of the main results is that IPS seems to effectively reduce time to getting a job compared with CAU interventions. These results also support the idea that time is a key element in the interventions. On the one hand, getting a job or getting back to work does not always depend on the individual’s capacity or interest. On the other hand, when people with serious mental disability are included in the sample, longer periods of employment support might be needed [58]. Indeed, welfare systems should play a role in the hatching of strategy implementations [59], for instance, in the differences between social insurance systems across countries. However, since IPS is based on personal interests, sometimes vocational rehabilitation initiatives include academic training, which could lengthen the time until the outcome is achieved. 

Previous reviews have already highlighted the importance of the services being close to each other or under the same roof between employment and mental health services [68], the communication between stakeholders [69], and direct and indirect costs related to reduced productivity [70]. Moreover, the achievement of a high-fidelity score within the IPS program appears to be essential for the effectiveness of the strategy [41]. Therefore, integration of employment services with the mental healthcare team should be a key element for the success of the interventions. In this sense, clinician training in facilitating employment strategies such as problem-solving, facing stress, and planning and scheduling work tasks might help improve the effectiveness of IPS treatment [41]. Some difficulties—such as the different methods, study design, heterogeneous samples, definition of outcome criteria, and diagnosis assessment—have also been previously reported [71,72,73].

In the educational field, another study highlighted a need to integrate the approaches to vocational guidance and support, as well as the importance of the educational and training needs in the workplace [74]. Similarly, training of clinicians in the multidisciplinary interventions has been proposed as a key element in mental health services [75]. In this sense, IPS appears to have the potential to enhance employment outcomes among those with MD, if policy initiatives and European governments support this program [75].

Evidence for RTW strategies remains unclear. Our results indicate that RTW strategies are mainly focused on employment factors, possibly neglecting powerful clinical variables and lessening the likelihood of success. Indeed, studies assessing PTSL showed limited effectiveness in reducing time to RTW for people with mental disorders. Since our selection criteria did not include clinical outcomes and not all the reviewed studies take them into account, we are not likely to determine whether these results represent a negative indicator. It may be that the strategy is not completely appropriate for MD, or perhaps participants using these strategies take more time to get back to work but do it with better stability (fewer relapse episodes), more satisfaction, and higher quality of life. In this sense, some participants could also benefit from a person-centered intervention [66].

Our review also suggests that the success of employment strategies is not always associated with better mental health status. Previous evidence has shown that even if working is not harmful to the mental health balance of people with severe mental disorders, nor does it seem to benefit this mental condition [27]. Not having a job has been shown to increase the probability of suffering from or aggravating physical health, stress, anxiety, and mood symptoms [76,77,78]. Additionally, not feeling healthy enough to get back to work may not have an impact in reduction of time until first return to work, whereas it seems to complicate full return to work. This evidence was found in one-to-one interventions [63,66] as well as web-based designed programs [64]. RTW strategies taking sickness symptoms and sickness absence into account seemed to slightly improve the effects of the intervention [52,55,57]. Moreover, case manager expertise [79], monitoring system prevention [55], and the alliance of clinical and work-focused treatments [52,53] may be considerable for employees back to work after a sick-leave period due to common mental disorders.

Previous RTW studies have demonstrated that case managers tend to select individuals better prepared for work, suggesting a selection bias that eventually affects effectiveness results [46]. Conversely, exposure-based strategies might hasten integration into the workforce [61], reducing the chances of the program being helpful in dealing with real experiences at work or work-related problems. The seriousness of the clinical illness symptoms seems, therefore, critical for the effectiveness of the intervention.

Evidence for the effectiveness impact of unemployment and disability benefits on employment integration remains unclear. Despite the fact that receiving these benefits might help people who are economically disadvantaged, it might also hinder the employment construction processes [80]. On the other hand, not receiving unemployment benefits could help people get a job sooner [62], increase employee income, and reduce the costs of allowances [32]. Building trust in people is considered necessary to work effectively in employment support services. However, fear of losing unemployment benefits, reduction of incomes, and benefit cutbacks could damage this trust [46].

Welfare benefits seem to be partially helpful in building employment projects for persons with MD. An alternative to long-term benefits could be to emphasize early return to work strategies and early interventions to job access, regardless of economic changes causing employment fluctuations.

Duration of the studies has been cited as a relevant factor [32,51,56,60]. Mean duration of the studies in the present review range from 1 to 2 years, in common with previous reviews. Therefore, longer follow-up periods are needed to extend the results of the successful strategies in the long-term run. Early return to work strategies seem to be essential, since feelings of anxiety grow, abilities decline, and perceived vulnerability increases with time needed to get back to work [23].

### Limitations

There are several limitations that should be highlighted. First, follow-up periods in different studies vary significantly, ranging from a minimum of 3 months to a maximum of 60 months. Second, the outcomes assessed in each study are quite heterogeneous, making comparison between studies difficult. Third, control groups might also be a source of bias, since the type of usual treatment might vary greatly from one setting to another. Fourth, different designs have been used across studies. Ideally, randomized controlled trials allow control for several confounders in an experimental approach (e.g., masking blinding participants, clinicians). However, within this complex field of research with multiple confounders, this is not always possible to prevent. Cohort studies are an alternative, naturalistic option, but results might lead to an overestimation of effectiveness. Additionally, the generalization of these results would be partially restricted due to the country of origin of the papers included (unrepresented western and southern Europe). Finally, our search could have been not sensitive enough to detect other emerging employment interventions, or they were excluded due to methodological reasons. Despite these limitations, our results contribute to extending the evidence on employment strategies in European countries, reporting a range of research designs and interventions and supporting and offering several recommendations in professional employment strategies for persons with MD. Moreover, the studies were found through the main research databases and the quality of the included papers was examined using relevant quality-control checklists.

## 5. Conclusions

Overall, the combination of clinician training in concrete job skills, case manager expertise, mental health tracking systems, and multidisciplinary interventions could help to improve the effectiveness of employment programs, which require intervention designs tailored to individual needs rather than organizational rules.

Future studies may improve research on the assessment of the effectiveness of employment interventions and also analyze the cost-effectiveness of such programs, taking into account direct and indirect aspects (sickness leave, replacements, medical service use, relapses, quality of life, self-confidence). These indirect costs are important to the employees, employers, and health care systems [52]. Since employment investments are needed to promote integration of persons with MD [81], SE programs should replace traditional interventions, leading to reduction in health, social, and economic costs.

## Figures and Tables

**Table 1:** Summary of the included European studies in review.

Authors, Year & Country	Name of Strategy	Type of Intervention Strategy	Employment Status Before Treatment	Sample (N, Mean Age, % Women)	Study Design	Study Period (Months)	Number of Follow-Up
Germundsson, P.; Gustafsson, J.; Lind, M.; Danermark, B. (2012) Sweden [32]	Vocational rehabilitation, according to the supported employment approach	Job access	Unemployed, not further described	*N* = 225 (46%)	Cohort	not clearly specified	2
Hogelund, J.; Holm, A.; Eplov, L. F. (2012) The Netherlands [66]	Part-time Sick Leave for Employees with Mental Disorders	RTW	Employed and on long-term sick leave	*N* = 226 (61%)	Cohort	18	0
van Veggel, R.; Waghorn, G. and Dias, S. (2015) UK [50]	Individual Placement and Support	Job access	Unemployed and seeking a job	*N* = 446 (39.6 years old, 44.7%)	Cohort	12	1
Andren, D. (2014) Sweden [51]	Part-time sick leave	RTW	Employed (open labor market) and on sick leave	Intervention groups (group 1: *N* = 548, 78%; group 2: *N* = 367, 73%; group 3: *N* = 172, 74%) Comparison groups (group 1: *N* = 79, 68%; group 2: *N* = 181, 66%; group 3: *N* = 155, 69%).	Cohort	Up to 12	12
Kroger, C.; Bode, K.; Wunsch, E. M.; Kliem, S.; Grocholewski, A.; Finger, F. (2014) Germany [52]	Psychotherapy intervention	RTW *Sickness absence*	Employed and on sick leave	Intervention group: *N* = 13 (38%). Comparison group: *N* = 13 (54%).	CT	12	1
Lagerveld, S. E.; Blonk, R. W.; Brenninkmeijer, V.; Wijngaards-de Meij, L.; Schaufeli, W. B. (2012) The Netherlands [53]	Work-Focused Treatment of Common Mental Disorders and Return to Work	RTW	Employed (open labor market) and on sick leave	*N* = 168 (40.7 years old, 60%) Intervention group: *N* = 89 (40.2 years old) Comparison group: *N* = 79 (41.3 years old)	CT	12	5
Martin, M. H.; Nielsen, M. B.; Madsen, I. E.; Petersen, S. M.; Lange, T.; Rugulies, R. (2013) Denmark [54]	Multidisciplinary, coordinated and tailored RTW intervention	RTW	Mix—employed and unemployed, sick-listed for 4–12 weeks	*N* = 76 (42 years old, 82%)	CT	3	1
Arends, I.; van der Klink, J. J.; van Rhenen, W.; de Boer, M. R.; Bultmann, U. (2014) The Netherlands [55]	Stimulating Healthy participation And Relapse Prevention (SHARP)-at work intervention	RTW *Sickness absence*	Employed (open labor market) who had sickness absence in the past	*N* = 158 (42.3 years old, 58.8%) Intervention group: *N* = 80. Comparison group: *N* = 78	RCT	12	3
Bejerholm, U.; Areberg, C.; Hofgren, C.; Sandlund, M.; Rinaldi, M. (2015) Sweden [56]	Individual Placement and Support	Job access	Unemployed and have a desire to work in the near future.	*N* = 120. Intervention group: *N* = 60 (53% females). Comparison group: *N* = 60 (35%)	RCT	18	2
Hees, H. L.; de Vries, G.; Koeter, M. W.; Schene, A. H. (2013) The Netherlands [57]	Occupational therapy, adjuvant to treatment as usual	RTW	Employed and on sick leave	*N* = 117 (42.6 years old) Intervention group: *N* = 39(47%) Comparison group: *N* = 78 (59%).	RCT	18	3
Heslin, M.; Howard, L.; Leese, M.; McCrone, P.; Rice, C.; Jarrett, M.; Spokes, T.; Huxley, P.; Thornicroft, G. (2011) UK [58]	Individual placement and support	Job access	Unemployed	*N* = 188 (not further described)	RCT	24	2
Hoffmann, Holger; Jäckel, Dorothea; Glauser, Sybille; Mueser, Kim T.; Kupper, Zeno (2012) Switzerland [59]	Supported employment	Job access	Not currently employed in the open market	Intervention group: *N* = 46 (33.5 years old), Comparison group: *N* = 54 (34.1 years old)	RCT	24	2
Hoffmann, H.; Jackel, D.; Glauser, S.; Kupper, Z. (2014) Switzerland [65]	Job Coach Project (Supported employment program)	Job access	Not currently employed in the open market	Intervention group: *N* = 46, Comparison group: *N* = 54 (33.5 years old, 35%)	RCT	60	2
Michon, H.; van Busschbach, J. T.; Stant, A. D.; van Vugt, M. D.; van Weeghel, J.; Kroon, H. (2014) The Netherlands [60]	Individual Placement and Support	Job access	Unemployed and seeking a job	Intervention group: *N* = 71 (34.1 years old, 27%) Comparison group: *N* = 80 (25%)	RCT	30	3
Noordik, E.; van der Klink, JJ.; Geskus, RB.; de Boer, MR.; van Dijk, FJ.; H. and Nieuwenhuijsen, K. (2013) The Netherlands [61]	Exposure-based return-to-work program for workers on sick leave due to common mental disorder	RTW	Employed (open labor market) and on sick leave	*N* = 160 (70.9%) Intervention group: *N* = 75 (44.9 years old) Comparison group: *N* = 85 (45.9 years old)	RCT	12	1
Reme, S. E.; Grasdal, A. L.; Lovvik, C.; Lie, S. A.; Overland, S. (2015) Norway [62]	AWaC (At Work and Coping)	Job status: RTW & Job access	Mix—people on and at risk of sick leave, and people on long-term benefits	Total sample: *N* = 1193 (40.4 years old). Intervention group: *N* = 437 (69.4%). Comparison group: *N* = 365 (65%)	RCT	12	1
Vlasveld, M. C.; van der Feltz-Cornelis, C. M.; Ader, H. J.; Anema, J. R.; Hoedeman, R.; van Mechelen, W.; Beekman, A. T. (2013) The Netherlands [63]	Collaborative care	RTW	Employed (open labor market) and on sick leave	Intervention group: *N* = 65 (41.9 years old) Comparison group: *N* = 51 (43.4 years old)	RCT	12	4
Volker, D., Zijlstra-Vlasveld, M. C., Anema, J. R., Beekman, A. T., Brouwers, E. P., Emons, W. H., van Lomwel, A. G. and van der Feltz-Cornelis, C. M. (2015) The Netherlands [64]	E-health module embedded in Collaborative Occupational health care	RTW	Employed (open labor market) and on sick leave	Intervention group: *N* = 131 (45.5 years old) Comparison group: *N* = 89 (45.5 years old)	RCT	12	4

RTW: return to work, CT: control trial, RCT: randomized control trial.

**Table 2:** Employment outcomes list, results, and references of the included review studies.

Reference	Employment Outcomes	Results	Quality Assessment
Germundsson, P.; et al. (2012) [32]	Obtaining a job: (1) level of employment; (2) disposable income; (3) sum of allowances.	The authors reported that supported employment participants were hired faster, earned a higher disposable income, and lower individual allowances. Significance was not reported.	+
Hogelund, J.; et al. (2012) [66]	Time to full RTW: (1) time until first return to regular working hours.	This study suggested that PTSL did not reduce duration until full RTW for employees with mental disorders. Without controlling unobserved characteristics, they found a strong and significant effect of PTSL for these employees with mental disorders. However, this effect disappeared after the correction for unobserved characteristics.	+
van Veggel, R.; et al. (2015) [50]	Competitive employment: (1) getting a job in competitive employment; (2) individuals accumulating 13 weeks or more employment; (3) individuals accumulating 26 weeks or more employment; (4) days to first job; (5) mean hours worked per week in employment.	The authors found that more IPS participants initiated competitive employment than pre-IPS participants (24.9% vs. 14.3%). Significance not reported.	+
Andren, D. (2014) [51]	Time to return to work: (1) fully recovering lost work capacity and (2) duration of sick leave.	This study suggests positive and significant effects of PTSL after 60 days of FTSL for persons with mental disorders.	+
Kroger, C.; et al. (2014) [52]	Sickness absence: (1) days of incapacity to work.	This study underlined that more W-CBT participants were working at the follow-up and the treatment effect size for W-CBT was significantly higher than the control group effect.	+
Lagerveld, S. E.; et al. (2012) [53]	RTW: (1) full RTW; (2) duration of full RTW; (3) duration of partial RTW; Process of RTW: (4) number of steps until full RTW; (5) RTW relapses.	The authors of the study found significant effects on duration until full RTW in the W-CBT group: full RTW occurred 65 days earlier and partial RTW occurred 12 days earlier. W-CBT experienced relapse more often, but the difference was not significant.	++
Martin, M. H.; et al. (2013) [54]	RTW: (1) time to RTW and (2) labor market status (self-supported, receiving sickness benefits, unemployment, disability, other).	This study found that the intervention significantly delayed time to RTW (HR = 0.50; 95% CI 0.34–0.75) in comparison with conventional case management.	+
Arends, I.; et al. (2014) [55]	Sickness absence: (1) recurrent sickness absence episodes; (2) time until recurrent sick absence.	This study underlined that the SHARP intervention was significantly effective in increasing the time until relapse and reducing sickness absence episodes, compared to care as usual.	+
Bejerholm, U.; et al. (2015) [56]	Competitive employment: (1) getting a job; (2) number of hours worked; (3) weeks worked; (4) job tenure; (5) income; and (6) time to first employment.	The authors found that IPS was significantly more effective than TVR in job access at 18-month follow-up (46% vs. 11%; difference 36%, 95% CI 18–54), as well as the number of working hours and weeks, longer job tenure periods, and income.	++
Hees, H. L.; et al. (2013) [57]	RTW: (1) time until partial RTW; (2) full RTW (3) absenteeism; (4) RTW with good health.	This study found that TAU+OT significantly accelerated work achievement and increased the probability of RTW in good health (GH). However, the addition of OT to TAU did not hasten recovery from depression.	++
Heslin, M.; et al. (2011) [58]	Job access: (1) competitive employment at 12 months; (2) competitive employment at 24 months	The authors of this IPS study reported that the intervention program was significantly more effective in obtaining a competitive job at 24 months follow-up than TAU (22% vs. 11%, *p* = 0.041). Previous work in the last 5 years also predicted job achievement and time to work attainment.	++
Hoffmann, H.; et al. (2012) [59]	Job access: (1) competitive employment rate; (2) length of employment at least 50% in competitive work (CW); (3) total weeks in CW; (4) annual weeks CW; (5) job tenure in longest CW held; (6) mean hours worked per year in CW; (7) cumulative duration of CW; (8) yearly income from CW; and (9) hourly competitive job wage in last 3 years.	This study showed that SE program was significantly more effective than TVR programs in assisting persons with severe mental illness to obtain and maintain competitive employment (65% compared with 33%).	++
Hoffmann, H.; et al. (2014) (Follow up study of Hoffmann et al., 2012) [65]	Job access: (1) competitive employment rate; (2) length of employment at least 50% in competitive work (CW); (3) total weeks in CW; (4) annual weeks CW; (5) job tenure in longest CW held; (6) mean hours worked per year in CW; (7) cumulative duration of CW; (8) yearly income from CW; and (9) hourly competitive job wage in last 3 years.	The authors found that SE intervention (IPS), at 5-year follow-up, was significantly more effective than TVR for competitive employment rate, length of employment, total weeks in CW, annual weeks CW, job tenure in longest CW, mean hours worked.	++
Michon, H.; et al. (2014) [60]	Rates of competitive employment: (1) gaining a competitive job; (2) days in competitive employment; (3) hours in competitive employment; (4) days to first job.	This study found that significantly more participants obtained competitive jobs before 18 and 30 months in the IPS group than the participants in the TVR group.	++
Noordik, E.; et al. (2013) [61]	RTW: (1) time to full RTW; (2) time to partial RTW; and (3) number of sick leave relapses.	The authors of this study reported that workers receiving the RTW-E intervention (209 days; 95% CI 62–256) had a significantly extended time to full RTW compared to workers receiving CAU (153 days; 95% CI 128–178).	+
Reme, S. E.; et al. (2015) [62]	Maintain or increase active work-life: (1) maintained work participation or new employment; (2) full or partial RTW.	This study showed that the intervention group had increased or maintained their work participation at follow-up compared to the control group (44.2% vs. 37.2%, *p* = 0.015). The effectiveness at 18 months remained significant. However, RTW results were inconsistent.	++
Vlasveld, M. C.; et al. (2013) [63]	RTW: (1) Duration until lasting, full RTW; (2) total number of sickness absence days	The results of this study suggested that the intervention was not significantly effective for the work-related outcomes. Collaborative care participants had a shorter time to response, with a difference of 2.8 months.	+
Volker, D.; et al. (2015) [64]	RTW: (1) time to first RTW; (2) time to full RTW; (3) number of days of sickness absence in the first-year follow-up.	The authors of the study determined inconclusive results. There was a significant RTW duration reduction until first RTW only. Time to full RTW and number of sickness absence days had no significant effects.	+

RTW: return to work, PTSL: part-time sick leave, IPS: individual placement and support, FTSL: full-time sick leave, W-CBT: work-focused cognitive-behavioral treatment, SHARP: stimulating healthy participation and relapse prevention, TVR: traditional vocational rehabilitation, TAU: treatment as usual, OT: occupational therapy, SE: supported employment, CAU: care as usual; RTW-E: exposure-based RTW.

## Supplementary Materials

The following are available online at http://www.mdpi.com/1660-4601/15/5/838/s1, the syntax extensive description, short description of each intervention program from the different studies included in this review, Figure S1: flowchart for the studies’ selection process.
