# Supplementary figures and images for: Furthering the Evidence of the Effectiveness of Employment Strategies for People with Mental Disorders in Europe: A Systematic Review
Source: Int J Environ Res Public Health. 2018 Apr 24;15(5):838. doi: 10.3390/ijerph15050838 (PMC5981877; doi:10.3390/ijerph15050838)

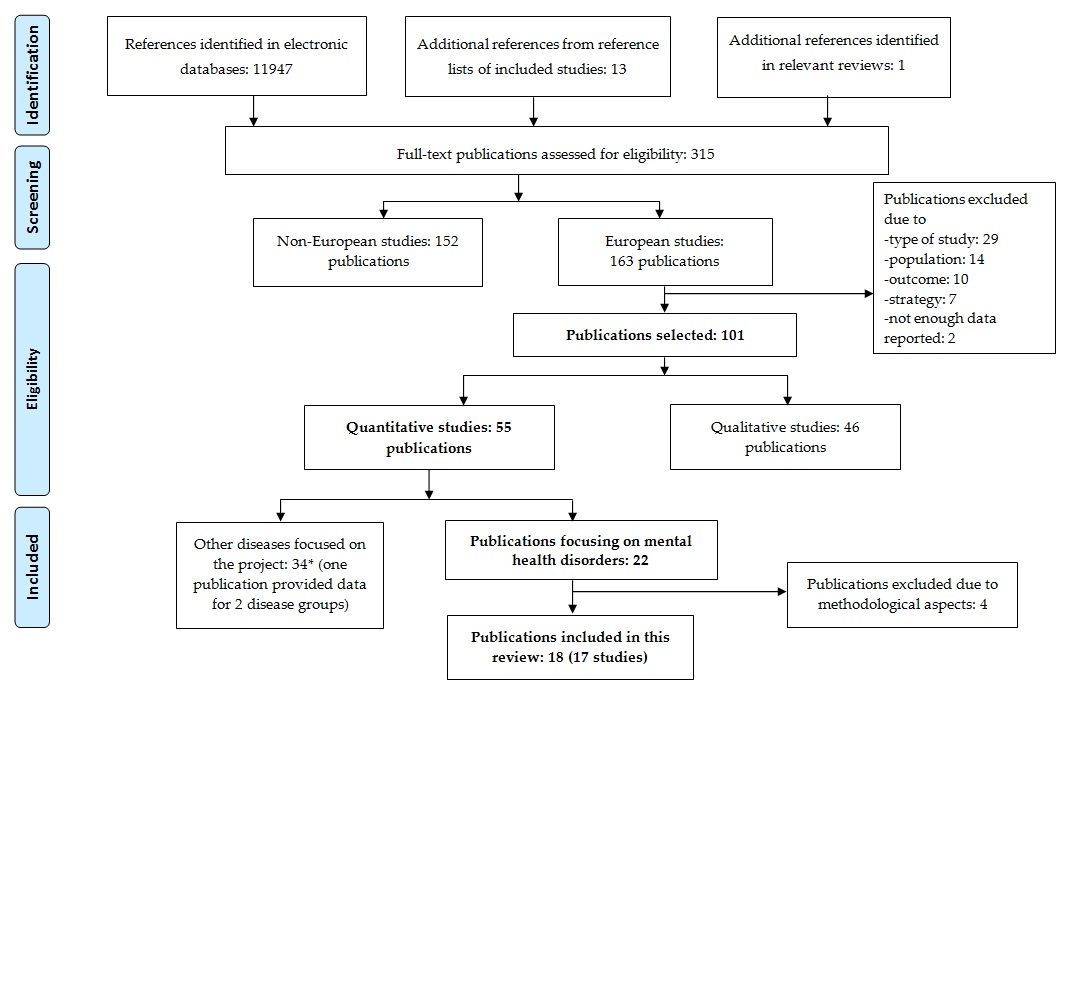

Supplement: Supplementary file 1 [file ijerph-15-00838-s001.zip › ijerph-284514-suppl/Figure S1. Flow chart description.jpg]
