# Supplementary material for: Furthering the Evidence of the Effectiveness of Employment Strategies for People with Mental Disorders in Europe: A Systematic Review
Source: Int J Environ Res Public Health. 2018 Apr 24;15(5):838. doi: 10.3390/ijerph15050838 (PMC5981877; doi:10.3390/ijerph15050838)
Supplement: Supplementary file 1 [file ijerph-15-00838-s001.zip › ijerph-284514-suppl/The short description.doc]

Furthering the Evidence of the effectiveness of employment strategies for people with mental health disorders in Europe: A systematic review

**DESCRIPTION OF THE INTERVENTIONS ASSESSED**

**Appendix B. Short description of each intervention program from the different studies included in this review.**

**1. Individual Placement and Support (IPS). The IPS Employment center (https://ipsworks.org/)**

The IPS approach is an evidence-based practice aiming to support persons with serious mental illness (e.g., bipolar disorder, depression, psychotic disorder). IPS program is a supported employment model assisting people in achieving a job in competitive employment. Other forms of career paths are also included in the program such as, education, training, tutoring, and public examinations.

IPS is based on 8 principles (Competitive employment, systematic job development, rapid job search, integrated services, benefits planning, zero exclusion, time-unlimited support, worker preferences).

**2. The Job Coach Project (https://doi.org/10.1186/ISRCTN26099032)**

The Job Coach Project was a study conducted in Switzerland. The aim of this IPS-approach study was to evaluate long-term effects of SE over 2 and 5 years.

Employment specialists having a maximum caseload of 15 participants, guided in a rapid job search based on the participant’s educational background, work preferences, and previous work experience. They had regular contact with the participants, at least once every two weeks and at least once a month with employment supervisors, clinicians or other professionals involved. Once the participants achieved employed, they received continuous training and support to enhance job preservation. Support and advocacy was provided in case of job loss.

**3. E-health module embedded in Collaborative Occupational health care (ECO) (http://www.trialregister.nl/trialreg/admin/rctview.asp?TC=2108)**

The ECO is a web-based intervention including 2 elements: First, the Return@Work eHealth module and second, an email decision aid for the occupational physician.

The ECO-intervention comprises detection of Major Depressive Disorder (MDD) amongst workers on sick leave by screening; web-based supporting diagnosis and treatment by occupational physicians (Ops); empowerment of the worker; collaborative and stepped care of MDD by evidence-based treatment recommendations and supported by a tracking system and consultant psychiatrist; relapse prevention; and work interventions, including consultation with employer and eventual work-accommodations.

These modules run in parallel to each other, and allow the occupational physicians to guide the employees, monitoring their symptoms, functioning and RTW.

**4. Occupational therapy (OT) adjuvant to treatment as usual (Hees HL, Koeter MW, de VG, et al. Effectiveness of adjuvant occupational therapy in employees with depression: design of a randomized controlled trial. BMC Public Health 2010;10:558)**

This 18-session intervention program focuses on three elements found to be essential for a successful RTW: an early return to work according to the ‘place-then-train’ principle, an increased focus on work-related coping and self-efficacy, and enhanced communication among the stakeholders involved.

The intervention program was conducted by two experienced and trained occupational therapists (OT). It is important to note that the program protocol included specific training for the OT as well as frequent communication between the OT and the corresponding psychiatrist. Additionally, participants were required to work at least 2 hours per week when starting OT to be able to directly practise the strategies learned during therapy.

**5. Part Time Sick Leave (PTSL) (Andrén, Daniela & Andrén, Thomas, 2008. "Part-Time Sick Leave as a Treatment Method? "Working Papers in Economics 320, University of Gothenburg, Department of Economics.)**

To facilitate a faster return to work, PTSL measure helps employees on full-time sick leave to work temporarily at reduced working hours. During PTSL, the employee receives the regular salary for the hours actively worked and a sickness benefit for the hours off work. Working hours increase whenever the employee’s health improves, to achieve full time working hours. In this RTW intervention, the employer and the employee reach consensus about the part time job contents and the municipal case manager must approve this agreement.

**6. Collaborative Care Treatment (Vlasveld MC, Anema JR, Beekman AT, van Mechelen W, Hoedeman R, Van Marwijk HW, et al. Multidisciplinary collaborative care for depressive disorder in the occupational health setting: design of a randomised controlled trial and cost-effectiveness study. BMC Health Serv Res 2008; 8: 99.)**

The collaborative care intervention is provided by the occupational physician case manager (OP-CM). It includes 6–12 sessions of Problem Solving Treatment (PST), manual-guided self-help, a workplace intervention and, depending on patient preference, prescription of antidepressant medication. PST is a brief, structured psychological intervention aimed at teaching the worker problem solving skills. Additionally, a self-help manual focused on cognitive restructuring is used, mainly addressing RTW and aspects of healthy lifestyle. The workplace intervention stage includes the assessment and work adjustments agreed by the worker, the OP-CM and the employer. In this sense, the employer and the employee both selected important obstacles for the RTW process, brainstormed for possible solutions and designed a pathway for implementation of the proposed solutions. The most important element in this intervention is reaching consensus about the RTW plan. Therefore, communication, participation and involvement of the employee and employer are essential.

**7. Work-Focused cognitive–behavioral therapy (W-CBT).( van Schie, J., Blonk, R. W. B., & Lagerveld, S. E. (2005). Werkhervatting bij arbeidsgerelateerde psychische klachten, behandelingsprotocol [Return to Work and work-related psychological complaints, treatment protocol]. Hoofddorp, The Netherlands: TNO.)**

W-CBT consisted of the regular cognitive–behavioral therapy (CBT) treatment plus a module focusing on work and the return to work process. The aim of this extended treatment of CBT is to change patterns of thinking or behave that hiders the ability to face situations and problems at work, for persons with mental health disorders.

Therapists conducted the intervention. The W-CBT treatment consisted first of regular CBT interventions which included specific work-related (homework) exercises/interventions. Second, all the interventions, exercises and homework were framed as much as possible in the work context. In addition, treatment time could also be spent on nonwork issues (e.g., marital problems). However, therapists were asked to relate these nonwork issues (at least partly) to work.

**8. RTW-E intervention (exposure-based return-to-work intervention) Noordik, E.; van der Klink, J. J.; Geskus, R. B.; de Boer, M. R.; van Dijk, F. J.; Nieuwenhuijsen, K. Effectiveness of an exposure-based return-to-work program for workers on sick leave due to common mental disorders: a cluster-randomized controlled trial. Scand. J. Work. Environ. Health 2013, 39, 144–154, doi:10.5271/sjweh.3320. TRIAL REGISTRATION: ISRCTN72643128.**

RTW-E is an exposure-based intervention program for workers on sick leave due to common mental disorders. The purpose of this program was to promote an active problem-solving behaviour in workers dealing with stressful work situations during return to work (RTW). In this sense, employees received care-as-usual (CAU) and were gradually exposed *in vivo* to a hierarchy of tasks representing more exigent work situations, including anxious, stressful or raging job settings.

Several protocol measures were considered: homework assignment, frequency of occupational physician consultations and reported communication of the employees with their supervisors.

**9. Multidisciplinary, coordinated and tailored RTW intervention (Martin, M. H. T.; Nielsen, M. B. D.; Madsen, I. E. H.; Petersen, S. M. A.; Lange, T.; Rugulies, R. Effectiveness of a Coordinated and Tailored Return-to-Work Intervention for Sickness Absence Beneficiaries with Mental Health Problems. J. Occup. Rehabil. 2013, 23, 621–630, doi:10.1007/s10926-013-9421-5.)**

This intervention based on RTW models, included different treatment stages: The first one was a screening assessment of work disability, conducted by a multidisciplinary team. The aim was to assess disability, functioning, barriers and resources for RTW in accordance with the International Classification of Functioning, Disability and Health. The second one was to develop an action plan for RTW, including proposed activities to overcome barriers and strengthen resources, and the third stage was related to the implementation of the action plan and regular updates according to the individual’s current situation.

**10. The SHARP-at work (Stimulating Healthy participation And Relapse Prevention), (Arends, I.; van der Klink, J. J. L.; van Rhenen, W.; de Boer, M. R.; Bültmann, U. Prevention of recurrent sickness absence in workers with common mental disorders: results of a cluster-randomised controlled trial. Occup. Environ. Med. 2014, 71, 21–29, doi:10.1136/oemed-2013-101412.)**

This evidence-based intervention delivered by occupational physicians aims to prevent recurrent sickness absence and encourage active participation for persons with common mental disorders.

The intervention consists of a five-step problem-solving process to identify and implement solutions for outlined difficulties in the RTW process. A channel of consultations was included between the worker and supervisor over the intervention process. The principal function of the occupational physician was to give counselling and assist the workers to categorize the problems and design their own solutions.

**11. The AWAC Project (At Work and Coping) (https://clinicaltrials.gov/ct2/show/NCT01146730)**

The AWAC is an innovative program, designed to integrate individual CBT and job support for persons with mental health disorders. Specific training was operated among the therapist and the employment specialist team to guarantee integration between CBT and work focus.

Up to 15 sessions of CBT were offered. Personalized job support was based on the IPS approach, developed for people with severe mental illness, and was offered in case of a need of individual job support (primarily participants with long-term disability) to enable job accommodation or identification of suitable employment.
