# Supplementary material for: Furthering the Evidence of the Effectiveness of Employment Strategies for People with Mental Disorders in Europe: A Systematic Review
Source: Int J Environ Res Public Health. 2018 Apr 24;15(5):838. doi: 10.3390/ijerph15050838 (PMC5981877; doi:10.3390/ijerph15050838)
Supplement: Supplementary file 1 [file ijerph-15-00838-s001.zip › ijerph-284514-suppl/The syntax extensive description.doc]

Furthering the Evidence of the effectiveness of employment strategies for people with mental health disorders in Europe: A systematic review

**SEARCH STRATEGIES**

**Database: Medline.**

**Interface: Ovid**

Search date: April 2016

| 1 | exp Disabled Persons/ |
| --- | --- |
| 2 | (disabled or disabilit*).mp. |
| 3 | Chronic Disease/ |
| 4 | (chronic ill* or chronic sick* or chronic absence* or chronic disease* or chronic condition*).mp. |
| 5 | (long standing sick* or long standing absence* or long standing disease* or long standing ill*).mp. |
| 6 | (longstanding sick* or longstanding absence* or longstanding disease* or longstanding ill*).mp. |
| 7 | llsi.ti,ab. |
| 8 | (long term sick* or long term absence* or long term disease* or long term ill*).mp. |
| 9 | (longterm sick* or longterm absence* or longterm disease* or longterm ill*).mp. |
| 10 | (permanent ill* or permanent absence* or permanent disease* or permanent sick*).mp. |
| 11 | Mental Disorders/ |
| 12 | (mental illness* or mental disorder* or mental health issue* or mental diagnosis or mental diagnoses or mental health problem* or mental health disorder* or psychological illness* or psychiatric illness* or psychological disorder* or psychiatric disorder* or psych* condition* or psych* diagnoses or psych* diagnosis or psych* problem*).ti,ab,kf. |
| 13 | depression.ti. |
| 14 | Depressive Disorder/ or Depressive Disorder, Major/ or Depression, Postpartum/ or Dysthymic Disorder/ |
| 15 | Nervous System Diseases/ |
| 16 | (Neurologic* disorder* or neurologic* disease* or nervous system disorder* or nervous system disease*).ti,ab,kf. |
| 17 | Headache Disorders/ or exp Headache Disorders, Primary/ |
| 18 | (Migraine* or headache*).ti,ab,kf. |
| 19 | Metabolic Disorders/ or Endocrine System Diseases/ |
| 20 | (metabolic disorder* or metabolic disease* or endocrine disorder* or endocrine disease*).ti,ab,kf. |
| 21 | Diabetes Mellitus/ or Diabetes Mellitus, Type 1/ or Diabetes Mellitus, Type 2/ |
| 22 | (diabetes or diabetic*).ab,kf,ti. |
| 23 | Musculoskeletal Diseases/ |
| 24 | (musculoskeletal disorder* or musculoskeletal disease* or musculoskeletal complaint* or musculoskeletal condition* or musculoskeletal problem* or musculoskeletal diagnosis or musculoskeletal diagnoses).ab,kf,ti. |
| 25 | exp Back Pain/ or Neck Pain/ |
| 26 | ((neck or cervical) and (pain or ache)).ab,kf,ti. |
| 27 | ((back or "spinal column") and (pain or ache)).ab,kf,ti. |
| 28 | (neckache or backache).ab,kf,ti. |
| 29 | Respiratory Tract Diseases/ |
| 30 | (respiratory illness* or respiratory disease* or respiratory disorder* or respiratory condition* or respiratory tract illness* or respiratory tract disease* or respiratory tract disorder* or respiratory tract indication* or respiratory tract condition* or respiratory tract diagnosis or respiratory tract diagnoses).ab,kf,ti. |
| 31 | exp Pulmonary Disease, Chronic Obstructive/ |
| 32 | (Chronic obstructive pulmonary disease or COPD or (bronch* and (disease* or disorder*) and chronic*) or pulmonary emphysema).ab,kf,ti. |
| 33 | Cardiovascular Diseases/ |
| 34 | (cardiovascular disorder* or cardiovascular disease*).ab,kf,ti. |
| 35 | Myocardial Ischemia/ or Myocardial Infarction/ |
| 36 | (ischemic heart disease or ischaemic heart disease or cardiac infarction or coronary infarction or heart attack or heart infarction). ab,kf,ti. |
| 37 | Neoplasms/ |
| 38 | (cancer* or carcinoma* or neoplasm* or sarcoma* or tumor*).ab,kf,ti. |
| 39 | 1 or 2 or 3 or 4 or 5 or 6 or 7 or 8 or 9 or 10 or 11 or 12 or 13 or 14 or 15 or 16 or 17 or 18 or 19 or 20 or 21 or 22 or 23 or 24 or 25 or 26 or 27 or 28 or 29 or 30 or 31 or 32 or 33 or 34 or 35 or 36 or 37 or 38 |
| 40 | Employment/ |
| 41 | exp Occupations/ |
| 42 | Work/ |
| 43 | Unemployment/ |
| 44 | Occupation or occupations.mp. |
| 45 | work*.mp. |
| 46 | vocation*.mp. |
| 47 | (unemploy* or employ*).mp. or (labour or labor).ti,ab. |
| 48 | (job or jobs).mp. |
| 49 | (earn* or paid or paying or payment*).mp. |
| 50 | jobseek*.mp. |
| 51 | Income/ or "Salaries and Fringe Benefits"/ |
| 52 | (salary or salari* or income or wages or waged or wage).mp. |
| 53 | 40 or 41 or 42 or 43 or 44 or 45 or 46 or 47 or 48 or 49 or 50 or 51 or 52 |
| 54 | rehabilitation/ or rehabilitation, vocational/ |
| 55 | Education, Professional, Retraining/ |
| 56 | welfare to work or welfare at work.mp. |
| 57 | (back to work or back into work).mp. |
| 58 | return to work.mp. |
| 59 | Training Support/ |
| 60 | training.mp. |
| 61 | retraining.mp. |
| 62 | re-training.mp. |
| 63 | (skill or skills).mp. |
| 64 | advice.mp. |
| 65 | Counseling/ |
| 66 | (counselling or counseling).mp. |
| 67 | Insurance, Disability/ |
| 68 | disability benefit*.mp. |
| 69 | Social Security/ |
| 70 | social security.mp. |
| 71 | Sick Leave/ |
| 72 | sick leave.mp. |
| 73 | Retirement/ |
| 74 | (mobility allowance* or mobility pension*).mp. |
| 75 | (disabilit* allowance* or disability* benefit*).mp. |
| 76 | sickness* benefit*.mp. |
| 77 | sickness* pension*or disability pension*.mp. |
| 78 | premature* retire*.mp. |
| 79 | early retire*.mp. |
| 80 | (quota or quotas).mp. |
| 81 | invalidity pension*.mp. |
| 82 | (invalidity allowance* or support allowance*).mp. |
| 83 | (invalidity benefit* or social benefit* or invalidity pension*).mp. |
| 84 | almp.ti,ab. or active labour market program*.mp. or active labor market program*.mp. |
| 85 | (employ* subsidy or employ* subsidies or wage subsidy or wage subsidies).mp. |
| 86 | (wage subsidy or wage subsidies).mp. |
| 87 | disability living allowance*.mp. |
| 88 | attendance allowance*.mp. |
| 89 | incapacity benefit*.mp. |
| 90 | incapacity allowance*.mp. |
| 91 | incapacity pension*.mp. |
| 92 | Employment, Supported/ |
| 93 | supported work.mp. |
| 94 | supported employ*.mp. |
| 95 | (tax incentive* or tax allowance* or tax credit*).mp. |
| 96 | work preparation*.mp. |
| 97 | Case Management/ |
| 98 | (Employment service* or case management).mp. |
| 99 | work focused interview*.mp. |
| 100 | (work trial or work trials).mp. |
| 101 | (employment trial or employment trials).mp. |
| 102 | (work placement* or job placement).mp. |
| 103 | (sheltered work* or sheltered employ*).mp. |
| 104 | (work preparation or job preparation).mp. |
| 105 | (workstep or work-step).mp. |
| 106 | (job match or workfare or work fare).mp. |
| 107 | access to work.mp. |
| 108 | jobmatch.mp. |
| 109 | (Worksite accommodation* or workplace accommodation* or work-place accommodation* or work accommodation* or job accommodation* or employment accommodation* or reasonable accommodation*).mp. |
| 110 | 54 or 55 or 56 or 57 or 58 or 59 or 60 or 61 or 62 or 63 or 64 or 65 or 66 or 67 or 68 or 69 or 70 or 71 or 72 or 73 or 74 or 75 or 76 or 77 or 78 or 79 or 80 or 81 or 82 or 83 or 84 or 85 or 86 or 87 or 88 or 89 or 90 or 91 or 92 or 93 or 94 or 95 or 96 or 97 or 98 or 99 or 100 or 101 or 102 or 103 or 104 or 105 or 106 or 107 or 108 or 109 |
| 111 | 39 and 53 and 110 |
| 112 | limit 111 to (abstracts and english language and yr="2011 - 2016") |

**Database: PSYCinfo**

**Interface: Ovid**

Search date: April 2016

| 1 | DE "Multiple Disabilities" OR DE "Disabilities" |
| --- | --- |
| 2 | DE "Chronic Illness" OR DE "Chronicity (Disorders)" |
| 3 | DE "Physical Disorders" AND (TI chronic or KW chronic) |
| 4 | TI (disabled or disabilit*or "chronic sick*" or "chronic ill*" or "chronic absence*" or "chronic disease*" or "chronic condition*") OR AB (disabled or disability* or "chronic sick*" or "chronic ill*" or "chronic absence*" or "chronic disease*" or "chronic condition*") OR DE (disabled or disability* or "chronic sick*" or "chronic ill*" or "chronic absence*" or "chronic disease*" or "chronic condition*") OR KW (disabled or disability* or "chronic sick*" or "chronic ill*" or "chronic absence*" or "chronic disease*" or "chronic condition*") |
| 5 | TI ("long standing sick*" or "long standing absence*" or "long standing disease*" or "long standing ill*") OR AB ("long standing sick*" or "long standing absence*" or "long standing disease*" or "long standing ill*") OR DE ("long standing sick*" or "long standing absence*" or "long standing disease*" or "long standing ill*") OR KW ("long standing sick*" or "long standing absence*" or "long standing disease*" or "long standing ill*") |
| 6 | TI ("longstanding sick*" or "longstanding absence*" or "longstanding disease*" or "longstanding ill*") OR AB ("longstanding sick*" or "longstanding absence*" or "longstanding disease*" or "longstanding ill*") OR DE ("longstanding sick*" or "longstanding absence*" or "longstanding disease*" or "longstanding ill*") OR KW ("longstanding sick*" or "longstanding absence*" or "longstanding disease*" or "longstanding ill*") |
| 7 | TI ("long term sick*" or "long term absence*" or "long term disease*" or "long term ill*") OR AB ("long term sick*" or "long term absence*" or "long term disease*" or "long term ill*") OR DE ("long term sick*" or "long term absence*" or "long term disease*" or "long term ill*") OR KW ("long term sick*" or "long term absence*" or "long term disease*" or "long term ill*") |
| 8 | TI ("permanent absence*" or "permanent disease*" or "permanent sick*" or "permanent ill*") OR AB ("permanent absence*" or "permanent disease*" or "permanent sick*" or "permanent ill*") OR DE ("permanent absence*" or "permanent disease*" or "permanent sick*" or "permanent ill*") OR KW ("permanent absence*" or "permanent disease*" or "permanent sick*" or "permanent ill*") |
| 9 | TI llsi OR AB llsi |
| 10 | DE "Chronic Mental Illness" |
| 11 | DE "Mental Disorders" |
| 12 | TI ("mental illness*" or "mental disorder*" or "mental health issue*" or "mental diagnosis" or "mental diagnoses" or "mental health problem*" or "mental health disorder*" or "psychological illness*" or "psychiatric illness*" or "psychological disorder*" or " psychiatric disorder*" or "psych* condition*" or "psych* diagnoses" or "psych* diagnosis" or "psych* problem*") OR AB ("mental illness*" or "mental disorder*" or "mental health issue*" or "mental diagnosis" or "mental diagnoses" or "mental health problem*" or "mental health disorder*" or "psychological illness*" or "psychiatric illness*" or "psychological disorder*" or " psychiatric disorder*" or "psych* condition*" or "psych* diagnoses" or "psych* diagnosis" or "psych* problem*") OR KW ("mental illness*" or "mental disorder*" or "mental health issue*" or "mental diagnosis" or "mental diagnoses" or "mental health problem*" or "mental health disorder*" or "psychological illness*" or "psychiatric illness*" or "psychological disorder*" or " psychiatric disorder*" or "psych* condition*" or "psych* diagnoses" or "psych* diagnosis" or "psych* problem*") |
| 13 | DE "Major Depression" OR DE "Dysthymic Disorder" OR DE "Endogenous Depression" OR DE "Postpartum Depression" OR DE "Reactive Depression" OR DE "Recurrent Depression" OR TI depression |
| 14 | DE "Nervous System Disorders" |
| 15 | TI ("Neurologic* disorder*" or "neurologic* disease*" or "nervous system disorder*" or "nervous system disease*") OR AB ("Neurologic* disorder*" or "neurologic* disease*" or "nervous system disorder*" or "nervous system disease*") OR KW ("Neurologic* disorder*" or "neurologic* disease*" or "nervous system disorder*" or "nervous system disease*") |
| 16 | DE "Headache" OR DE "Migraine Headache" OR DE "Muscle Contraction Headache" |
| 17 | TI (migraine or headache) OR AB (migraine or headache) OR KW (migraine or headache) |
| 18 | DE "Metabolism Disorders" OR DE "Endocrine Disorders" |
| 19 | TI ("metabolic disorder*" or "metabolic disease*" or "endocrine disorder*" or "endocrine disease*") OR AB ("metabolic disorder*" or "metabolic disease*" or "endocrine disorder*" or "endocrine disease*") OR KW ("metabolic disorder*" or "metabolic disease*" or "endocrine disorder*" or "endocrine disease*") |
| 20 | DE "Diabetes" OR DE "Diabetes Mellitus" OR DE "Type 2 Diabetes" |
| 21 | TI (diabetes or diabetic*) OR AB (diabetes or diabetic*) OR KW (diabetes or diabetic*) |
| 22 | DE "Musculoskeletal Disorders" |
| 23 | TI ("musculoskeletal disorder*" or "musculoskeletal disease*" or "musculoskeletal complaint*" or "musculoskeletal condition*" or "musculoskeletal problem*" or "musculoskeletal diagnosis" or "musculoskeletal diagnoses") OR AB ("musculoskeletal disorder*" or "musculoskeletal disease*" or "musculoskeletal complaint*" or "musculoskeletal condition*" or "musculoskeletal problem*" or "musculoskeletal diagnosis" or "musculoskeletal diagnoses") OR KW ("musculoskeletal disorder*" or "musculoskeletal disease*" or "musculoskeletal complaint*" or "musculoskeletal condition*" or "musculoskeletal problem*" or "musculoskeletal diagnosis" or "musculoskeletal diagnoses") |
| 24 | DE "Back Pain" |
| 25 | TI ((neck or cervical) and (pain or ache)) OR AB ((neck or cervical) and (pain or ache)) OR KW ((neck or cervical) and (pain or ache)) |
| 26 | TI ((back or "spinal column") and (pain or ache)) |
| 27 | TI (neckache or backache) |
| 28 | DE "Respiratory Tract Disorders" |
| 29 | TI ("respiratory illness*" or "respiratory disease*" or "respiratory disorder*" or "respiratory condition*" or "respiratory tract illness*" or "respiratory tract disease*" or "respiratory tract disorder*" or "respiratory tract indication*" or "respiratory tract condition*" or "respiratory tract diagnosis" or "respiratory tract diagnoses") OR AB ("respiratory illness*" or "respiratory disease*" or "respiratory disorder*" or "respiratory condition*" or "respiratory tract illness*" or "respiratory tract disease*" or "respiratory tract disorder*" or "respiratory tract indication*" or "respiratory tract condition*" or "respiratory tract diagnosis" or "respiratory tract diagnoses") OR KW ("respiratory illness*" or "respiratory disease*" or "respiratory disorder*" or "respiratory condition*" or "respiratory tract illness*" or "respiratory tract disease*" or "respiratory tract disorder*" or "respiratory tract indication*" or "respiratory tract condition*" or "respiratory tract diagnosis" or "respiratory tract diagnoses") |
| 30 | DE "Chronic Obstructive Pulmonary Disease" OR DE "Bronchial Disorders" OR DE "Pulmonary Emphysema" |
| 31 | TI ("Chronic obstructive pulmonary disease" or COPD or (bronch* and (disease* or disorder*) and chronic*) or "pulmonary emphysema") OR AB ("Chronic obstructive pulmonary disease" or COPD or (bronch* and (disease* or disorder*) and chronic*) or "pulmonary emphysema") OR KW ("Chronic obstructive pulmonary disease" or COPD or (bronch* and (disease* or disorder*) and chronic*) or "pulmonary emphysema") |
| 32 | DE "Cardiovascular disorders" |
| 33 | TI ("cardiovascular disorder*" or "cardiovascular disease*") or AB ("cardiovascular disorder*" or "cardiovascular disease*") OR KW ("cardiovascular disorder*" or "cardiovascular disease*") |
| 34 | DE "Myocardial Infarctions" |
| 35 | TI ("ischemic heart disease" or "ischaemic heart disease" or "cardiac infarction" or "coronary infarction" or "heart attack" or "heart infarction") OR AB ("ischemic heart disease" or "ischaemic heart disease" or "cardiac infarction" or "coronary infarction" or "heart attack" or "heart infarction") OR KW ("ischemic heart disease" or "ischaemic heart disease" or "cardiac infarction" or "coronary infarction" or "heart attack" or "heart infarction") |
| 36 | DE "Neoplasms" |
| 37 | TI (cancer* or carcinoma* or neoplasm* or sarcoma* or tumor*) OR AB (cancer* or carcinoma* or neoplasm* or sarcoma* or tumor*) OR DE (cancer* or carcinoma* or neoplasm* or sarcoma* or tumor*) OR KW (cancer* or carcinoma* or neoplasm* or sarcoma* or tumor*) |
| 38 | S1 OR S2 OR S3 OR S4 OR S5 OR S6 OR S7 OR S8 OR S9 OR S10 OR S11 OR S12 OR S13 OR S14 OR S15 OR S16 OR S17 OR S18 OR S19 OR S20 OR S21 OR S22 OR S23 OR S24 OR S25 OR S26 OR S27 OR S28 OR S29 OR S30 OR S31 OR S32 OR S33 OR S34 OR S35 OR S36 OR S37 |
| 39 | DE "Employment Status" OR DE "Self-Employment" OR DE "Unemployment" OR DE "Employability" |
| 40 | DE "Occupations" OR DE "Nontraditional Careers" OR DE "Occupational Status" |
| 41 | DE "Income Level" OR DE "Lower Income Level" OR DE "Middle Income Level" OR DE "Upper Income Level" |
| 42 | DE "Salaries" |
| 43 | TI (occupation or occupations or work* or vocation* or job or jobs or jobseek* or earn* or paid or paying or payment*) OR AB (occupation or occupations or work* or vocation* or job or jobs or jobseek* or earn* or paid or paying or payment*) OR KW (occupation or occupations or work* or vocation* or job or jobs or jobseek* or earn* or paid or paying or payment*) |
| 44 | TI (salary or salaries or income or wages or waged or wage or unemploy* or employ*) OR AB (salary or salaries or income or wages or waged or wage or unemploy* or employ*) OR KW (salary or salaries or income or wages or waged or wage or unemploy* or employ*) |
| 45 | TI (labour or labor) or AB (labour or labor) OR KW (labour or labor) |
| 46 | S39 OR S40 OR S41 OR S42 OR S43 OR S44 OR S45 |
| 47 | DE "Rehabilitation" OR DE "Cognitive Rehabilitation" OR DE "Criminal Rehabilitation" OR DE "Drug Rehabilitation" OR DE "Neuropsychological Rehabilitation" OR DE "Neurorehabilitation" OR DE "Occupational Therapy" OR DE "Physical Therapy" OR DE "Psychosocial Rehabilitation" |
| 48 | DE "Vocational rehabilitation" OR DE "Supported employment" OR "Vocational evaluation" OR DE "Work adjustment training" OR DE "Disability management" OR DE “Rehabilitation counseling" |
| 49 | DE "On the Job Training" |
| 50 | DE "Disability Laws" |
| 51 | DE "Vocational Education" OR DE "Cooperative Education" |
| 52 | DE "Retirement" OR DE "Reemployment" |
| 53 | DE "Counseling" |
| 54 | DE "Social Security" OR DE "Disability Evaluation" |
| 55 | DE "Employee Leave Benefits" |
| 56 | TI ("welfare to work" or "welfare at work") OR AB ("welfare to work" or "welfare at work") OR DE ("welfare to work" or "welfare at work") OR KW ("welfare to work" or "welfare at work") |
| 57 | TI ("back to work" or "back into work") OR AB ("back to work" or "back into work") OR DE ("back to work" or "back into work") OR KW ("back to work" or "back into work") |
| 58 | TI ("sick leave" or "disability benefit*" or "disability pension*" or training or retraining or re-training or skill or skills or advice or counselling or counseling) OR AB ("sick leave" or "disability benefit*" or "disability pension*" or training or retraining or re-training or skill or skills or advice or counselling or counseling) OR DE ("sick leave" or disability benefit* or disability pension* or training or retraining or re-training or skill or skills or advice or counselling or counseling) OR KW ("sick leave" or "disability benefit*" or "disability pension*" or training or retraining or re-training or skill or skills or advice or counselling or counseling) |
| 59 | TI (quota or quotas or "mobility pension*" or "invalidity pension*" or "invalidity allowance*" or "invalidity benefit*" or "social benefit*" or "support allowance*") OR AB (quota or quotas or "mobility pension*" or "invalidity pension*" or "invalidity allowance*" or "invalidity benefit*" or "social benefit*" or "support allowance*") OR DE (quota or quotas or "mobility pension*" or "invalidity pension*" or "invalidity allowance*" or "invalidity benefit*" or "social benefit*" or "support allowance*") OR KW (quota or quotas or "mobility pension*" or "invalidity pension*" or "invalidity allowance*" or "invalidity benefit*" or "social benefit*" or "support allowance*") |
| 60 | TI ("disability living allowance*" or "attendance allowance*" or "incapacity benefit*" or "incapacity allowance*" or "incapacity pension*") OR AB ("disability living allowance*" or "attendance allowance*" or "incapacity benefit*" or "incapacity allowance*" or "incapacity pension*") OR DE ("disability living allowance*" or "attendance allowance*" or "incapacity benefit*" or "incapacity allowance*" or "incapacity pension*") OR KW ("disability living allowance*" or "attendance allowance*" or "incapacity benefit*" or "incapacity allowance*" or "incapacity pension*") |
| 61 | TI ("employ* subsidy" or "employ* subsidies" or "wage subsidy" or "wage subsidies" or "tax incentive*" or "tax allowance*" or "tax credit*" or "social security") OR AB ("employ* subsidy" or "employ* subsidies" or "wage subsidy" or "wage subsidies" or "tax incentive*" or "tax allowance*" or "tax credit*" or "social security") OR DE ("employ* subsidy" or "employ* subsidies" or "wage subsidy" or "wage subsidies" or "tax incentive*" or "tax allowance*" or "tax credit*" or "social security") OR KW ("employ* subsidy" or "employ* subsidies" or "wage subsidy" or "wage subsidies" or "tax incentive*" or "tax allowance*" or "tax credit*" or "social security") |
| 62 | TI (almp) OR AB (almp) OR TI ("active labour market program*" or "active labor market program*") OR AB ("active labour market program*" or "active labor market program*") OR DE ("active labour market program*" or "active labor market program*") OR KW ("active labour market program*" or "active labor market program*") |
| 63 | TI ("supported work or supported employ*") OR AB ("supported work or supported employ*") OR DE ("supported work or supported employ*") OR KW ("supported work or supported employ*") |
| 64 | TI ("work placement*" or "job placement*" or "employment service*" or "case management" or "work focused interview*") OR AB ("work placement*" or "job placement*" or "employment service*" or "case management" or "work focused interview*") OR DE ("work placement*" or "job placement*" or "employment service*" or "case management" or "work focused interview*") OR KW ("work placement*" or "job placement*" or "employment service*" or "case management" or "work focused interview*") |
| 65 | TI ("work preparation*" or "job preparation*" or "employment trial" or "employment trials" or "work trial" or "work trials" or workstep or work-step) OR AB ("work preparation*" or "job preparation*" or "employment trial" or "employment trials" or "work trial" or "work trials" or workstep or work-step) OR DE ("work preparation*" or "job preparation*" or "employment trial" or "employment trials" or "work trial" or "work trials" or workstep or work-step) OR KW ("work preparation*" or "job preparation*" or "employment trial" or "employment trials" or "work trial" or "work trials" or workstep or work-step) |
| 66 | TI ("job match" or workfare or "work fare" or "access to work") OR AB ("job match" or workfare or "work fare" or "access to work") OR DE ("job match" or workfare or "work fare" or "access to work") OR KW ("job match" or workfare or "work fare" or "access to work") |
| 67 | TI ("sheltered work*" or "sheltered employ*") OR AB ("sheltered work*" or "sheltered employ*") OR DE ("sheltered work*" or "sheltered employ*") OR KW ("sheltered work*" or "sheltered employ*") |
| 68 | TI ("worksite accommodation*" or "workplace accommodation*" or "work-place accommodation*" or "work accommodation*" or "job accommodation*" or "employment accommodation*" or "reasonable accommodation*") OR AB ("worksite accommodation*" or "workplace accommodation*" or "work-place accommodation*" or "work accommodation*" or "job accommodation*" or "employment accommodation*" or "reasonable accommodation*") OR DE ("worksite accommodation*" or "workplace accommodation*" or "work-place accommodation*" or "work accommodation*" or "job accommodation*" or "employment accommodation*" or "reasonable accommodation*") OR KW ("worksite accommodation*" or "workplace accommodation*" or "work-place accommodation*" or "work accommodation*" or "job accommodation*" or "employment accommodation*" or "reasonable accommodation*") |
| 69 | DE "Accommodation (Disabilities)" |
| 70 | TI (Strategy or strategies or intervention* or program or programs or programme or programmes) |
| 71 | S47 OR S48 OR S49 OR S50 OR S51 OR S52 OR S53 OR S54 OR S55 OR S56 OR S57 OR S58 OR S59 OR S60 OR S61 OR S62 OR S63 OR S64 OR S65 OR S66 OR S67 OR S68 OR S69 OR 70 |
| 72 | S38 AND S46 AND S71 (Limits: English, peer-reviewed, 2011-2016) |

**Databases: Cochrane Reviews, Other Reviews and Health Technology Assessment**

**Interface: Ovid**

Search date: April 2016

| 1 | MeSH descriptor: [Disabled Persons] explode all trees |
| --- | --- |
| 2 | MeSH descriptor: [Chronic Disease] this term only |
| 3 | disabled or disabilit* or (chronic next sick*) or (chronic next ill*) or (chronic next absence*) or (chronic next disease*):ti,ab,kw (Word variations have been searched) |
| 4 | (long next standing next ill*) or (long next standing next sick*) or (long next standing next absence*) or (long next standing next disease*):ti,ab,kw (Word variations have been searched) |
| 5 | (longstanding next sick*) or (longstanding next ill*) or (longstanding next absence*) or (longstanding next disease*) or llsi:ti,ab,kw (Word variations have been searched) |
| 6 | (long next term next sick*) or (long next term next absence*) or (long next term next disease*):ti,ab,kw (Word variations have been searched) |
| 7 | (long next term next ill*) or (longterm next ill*) or (longterm next sick*) or (longterm next absence*) or (longterm next disease*):ti,ab,kw (Word variations have been searched) |
| 8 | (permanent next sick*) or (permanent next ill*) or (permanent next absence*) or (permanent next disease*):ti,ab,kw (Word variations have been searched) |
| 9 | (#1 or #2 or #3 or #4 or #5 or #6 or #7 or #8) |
| 10 | MeSH descriptor: [Employment] this term only |
| 11 | MeSH descriptor: [Occupations] explode all trees |
| 12 | MeSH descriptor: [Work] this term only |
| 13 | MeSH descriptor: [Unemployment] explode all trees |
| 14 | MeSH descriptor: [Income] this term only |
| 15 | MeSH descriptor: [Salaries and Fringe Benefits] this term only |
| 16 | (Occupation or occupations or work* or vocation* or job or jobs or earn* or paid or paying or payment*):ti,ab,kw (Word variations have been searched) |
| 17 | (salary or salari* or income or wages or waged or wage or unemploy* or employ* or labour or labor):ti,ab,kw (Word variations have been searched) |
| 18 | (#10 or #11 or #12 or #13 or #14 or #15 or #16 or #17) |
| 19 | MeSH descriptor: [Rehabilitation] this term only |
| 20 | MeSH descriptor: [Rehabilitation, Vocational] this term only |
| 21 | MeSH descriptor: [Education, Professional, Retraining] this term only |
| 22 | MeSH descriptor: [Training Support] this term only |
| 23 | MeSH descriptor: [Counseling] this term only |
| 24 | MeSH descriptor: [Insurance, Disability] this term only |
| 25 | MeSH descriptor: [Insurance, Liability] this term only |
| 26 | MeSH descriptor: [Social Security] explode all trees |
| 27 | MeSH descriptor: [Sick Leave] this term only |
| 28 | MeSH descriptor: [Retirement] this term only |
| 29 | MeSH descriptor: [Employment, Supported] this term only |
| 30 | "welfare to work" or "welfare at work" or "back to work" or "back into work":ti,ab,kw (Word variations have been searched) |
| 31 | (sick next leave) or (disability next benefit*) or (disability next pension*) or training or retraining or (re next training) or skill or skills or advice or counselling or counseling:ti,ab,kw (Word variations have been searched) |
| 32 | (mobility next allowance*) or (disabilit* next allowance*) or (sickness* next benefit*) or (sickness* next pension*) or (premature* next retire*) or (early next retire*):ti,ab,kw (Word variations have been searched) |
| 33 | (quota or (mobility next pension*) or (invalidity next pension*) or (invalidity next allowance*) or (invalidity next benefit*) or quotas or (employ* next subsidy) or (employ* next subsidies)):ti,ab,kw (Word variations have been searched) |
| 34 | (disability next living next allowance*) or (attendance next allowance*) or (incapacity next benefit*) or (incapacity next allowance*) or (incapacity next pension*) or (severe next disablement next allowance*):ti,ab,kw (Word variations have been searched) |
| 35 | (supported next work) or (supported next employ*) or (disabled next persons next tax next allowance*) or (access next to next work next program*):ti,ab,kw (Word variations have been searched) |
| 36 | ((work next preparation*) or (work next focussed next interview*) or (work next focused next interview*) or workstep or (work next step)):ti,ab,kw (Word variations have been searched) |
| 37 | (disability next working next allowance*) or (condition next management next program*) or "work trial" or "work trials":ti,ab,kw (Word variations have been searched) |
| 38 | "employment trial" or "employment trials" or (work next placement*) or (disability next discrimination next act) or (work next preparation):ti,ab,kw (Word variations have been searched) |
| 39 | (job next preparation) or (work next preparation) or (job next placement) or (work next placement) or (job next match) or (employment next service*) or (case next management) or workfare or (work next fare):ti,ab,kw (Word variations have been searched) |
| 40 | "access to work" or jobmatch or (active next labour next market next program*) or (active next labor next market next program*) or almp or (worksite next accommodation*) or (workplace next accommodation*) or (work-place next accommodation*) or (work next accommodation*) or (job next accommodation*) or (employment next accommodation*) or (reasonable next accommodation*):ti,ab,kw (Word variations have been searched) |
| 41 | (#19 or #20 or #21 or #22 or #23 or #24 or #25 or #26 or #27 or #28 or #29 or #30 or #31 or #32 or #33 or #34 or #35 or #36 or #37 or #38 or #39 or #40) |
| 42 | (#9 and #18 and #41) |
| 43 | MeSH descriptor: [Mental Disorders] this term only |
| 44 | (mental illness*) or (mental disorder*) or (mental health issue*) or (mental diagnosis) or (mental diagnoses) or (mental health problem*) or (mental health disorder*) or (psychological illness*) or (psychiatric illness*) or (psychological disorder*) or (psychiatric disorder*) or (psych* condition*) or (psych* diagnoses) or (psych* diagnosis) or (psych* problem*):ti,ab,kw (Word variations have been searched) |
| 45 | depression:ti (Word variations have been searched) |
| 46 | MeSH descriptor: [Depressive Disorder] this term only |
| 47 | MeSH descriptor: [Depressive Disorder, Major] this term only |
| 48 | MeSH descriptor: [Nervous System Diseases] this term only |
| 49 | (Neurologic* disorder*) or (neurologic* disease*) or (nervous system disorder*) or (nervous system disease*):ti,ab,kw (Word variations have been searched) |
| 50 | MeSH descriptor: [Headache Disorders] this term only |
| 51 | MeSH descriptor: [Headache Disorders, Primary] explode all trees |
| 52 | (Migraine*) or (headache*):ti,ab,kw (Word variations have been searched) |
| 53 | MeSH descriptor: [Metabolic Diseases] this term only |
| 54 | MeSH descriptor: [Endocrine System Diseases] this term only |
| 55 | (metabolic disorder) * or (metabolic disease*) or (endocrine disorder*) or (endocrine disease*):ti,ab,kw (Word variations have been searched) |
| 56 | MeSH descriptor: [Diabetes Mellitus] this term only |
| 57 | MeSH descriptor: [Diabetes Mellitus, Type 1] this term only |
| 58 | MeSH descriptor: [Diabetes Mellitus, Type 2] this term only |
| 59 | (diabetes) or (diabetic*):ti,ab,kw (Word variations have been searched) |
| 60 | MeSH descriptor: [Musculoskeletal Diseases] this term only |
| 61 | (musculoskeletal disorder*) or (musculoskeletal disease*) or (musculoskeletal complaint*) or (musculoskeletal condition*) or (musculoskeletal problem*) or (musculoskeletal diagnosis) or (musculoskeletal diagnoses):ti,ab,kw (Word variations have been searched) |
| 62 | MeSH descriptor: [Neck Pain] this term only |
| 63 | MeSH descriptor: [Back Pain] explode all trees |
| 64 | (neck or cervical) and (pain or ache):ti,ab,kw (Word variations have been searched) |
| 65 | (back or "spinal column") and (pain or ache):ti,ab,kw (Word variations have been searched) |
| 66 | (neckache) or (backache):ti,ab,kw (Word variations have been searched) |
| 67 | MeSH descriptor: [Respiratory Tract Diseases] this term only |
| 68 | (respiratory illness*) or (respiratory disease*) or (respiratory disorder*) or (respiratory condition*) or (respiratory tract illness*) or (respiratory tract disease*) or (respiratory tract disorder*) or (respiratory tract indication*) or (respiratory tract condition*) or (respiratory tract diagnosis) or (respiratory tract diagnoses):ti,ab,kw (Word variations have been searched) |
| 69 | MeSH descriptor: [Pulmonary Disease, Chronic Obstructive] explode all trees |
| 70 | (Chronic obstructive pulmonary disease) or COPD or (bronch* next (disease* or disorder*) next chronic*) or (pulmonary emphysema):ti,ab,kw (Word variations have been searched) |
| 71 | MeSH descriptor: [Cardiovascular Diseases] this term only |
| 72 | (cardiovascular disorder*) or (cardiovascular disease*):ti,ab,kw (Word variations have been searched) |
| 73 | MeSH descriptor: [Myocardial Ischemia] explode all trees |
| 74 | (ischemic heart disease) or (ischaemic heart disease) or (cardiac infarction) or (coronary infarction) or (heart attack) or (heart infarction):ti,ab,kw (Word variations have been searched) |
| 75 | MeSH descriptor: [Neoplasms] this term only |
| 76 | (cancer*) or (carcinoma*) or (neoplasm*) or (sarcoma*) or (tumor*):ti,ab,kw (Word variations have been searched) |
| 77 | (#43 or #44 or #45 or #46 or #47 or #48 or #49 or #50 or #51 or #52 or #53 or #54 or #55 or #56 or #57 or #58 or #59 or #60 or #61 or #62 or #63 or #64 or #65 or #66 or #67 or #68 or #69 or #70 or #71 or #72 or #73 or #74 or #75 or #76) |
| 78 | (#9 or #77) |
| 79 | (#78 and #18 and #41)  Online Publication Date from Jan 2011 to Apr 2016, in Cochrane Reviews (Reviews only) |
| 81 | (#78 and #18 and #41)  Publication Year from 2011 to 2016, in Other Reviews |
| 82 | (#78 and #18 and #41)  Publication Year from 2011 to 2016, in Health Technology Assessment |
